# Polysaccharide-Based Hydrogels for Microencapsulation of Stem Cells in Regenerative Medicine

**DOI:** 10.3389/fbioe.2021.735090

**Published:** 2021-10-18

**Authors:** Si-Yuen Lee, Jingyi Ma, Tze Sean Khoo, Norfadhilatuladha Abdullah, Nik Nur Farisha Nik Md Noordin Kahar, Zuratul Ain Abdul Hamid, Muzaimi Mustapha

**Affiliations:** ^1^ Department of Medicine, School of Medical Sciences, Universiti Sains Malaysia, Kota Bharu, Malaysia; ^2^ Duke-NUS Medical School, Singapore, Singapore; ^3^ UKM Medical Molecular Biology Institute, National University of Malaysia, Bangi, Malaysia; ^4^ Advanced Membrane Technology Research Centre, Faculty of Chemical and Energy Engineering, Universiti Teknologi Malaysia, Skudai, Malaysia; ^5^ School of Materials and Mineral Resources Engineering, Universiti Sains Malaysia, Nibong Tebal, Malaysia; ^6^ Department of Neurosciences, School of Medical Sciences, Universiti Sains Malaysia, Kota Bharu, Malaysia

**Keywords:** polysaccharide hydrogels, stem cells, microencapsulation, regenerative medicine, cell delivery, disease modeling

## Abstract

Stem cell-based therapy appears as a promising strategy to induce regeneration of damaged and diseased tissues. However, low survival, poor engraftment and a lack of site-specificity are major drawbacks. Polysaccharide hydrogels can address these issues and offer several advantages as cell delivery vehicles. They have become very popular due to their unique properties such as high-water content, biocompatibility, biodegradability and flexibility. Polysaccharide polymers can be physically or chemically crosslinked to construct biomimetic hydrogels. Their resemblance to living tissues mimics the native three-dimensional extracellular matrix and supports stem cell survival, proliferation and differentiation. Given the intricate nature of communication between hydrogels and stem cells, understanding their interaction is crucial. Cells are incorporated with polysaccharide hydrogels using various microencapsulation techniques, allowing generation of more relevant models and further enhancement of stem cell therapies. This paper provides a comprehensive review of human stem cells and polysaccharide hydrogels most used in regenerative medicine. The recent and advanced stem cell microencapsulation techniques, which include extrusion, emulsion, lithography, microfluidics, superhydrophobic surfaces and bioprinting, are described. This review also discusses current progress in clinical translation of stem-cell encapsulated polysaccharide hydrogels for cell delivery and disease modeling (drug testing and discovery) with focuses on musculoskeletal, nervous, cardiac and cancerous tissues.

## Introduction

**FIGURE 1:**
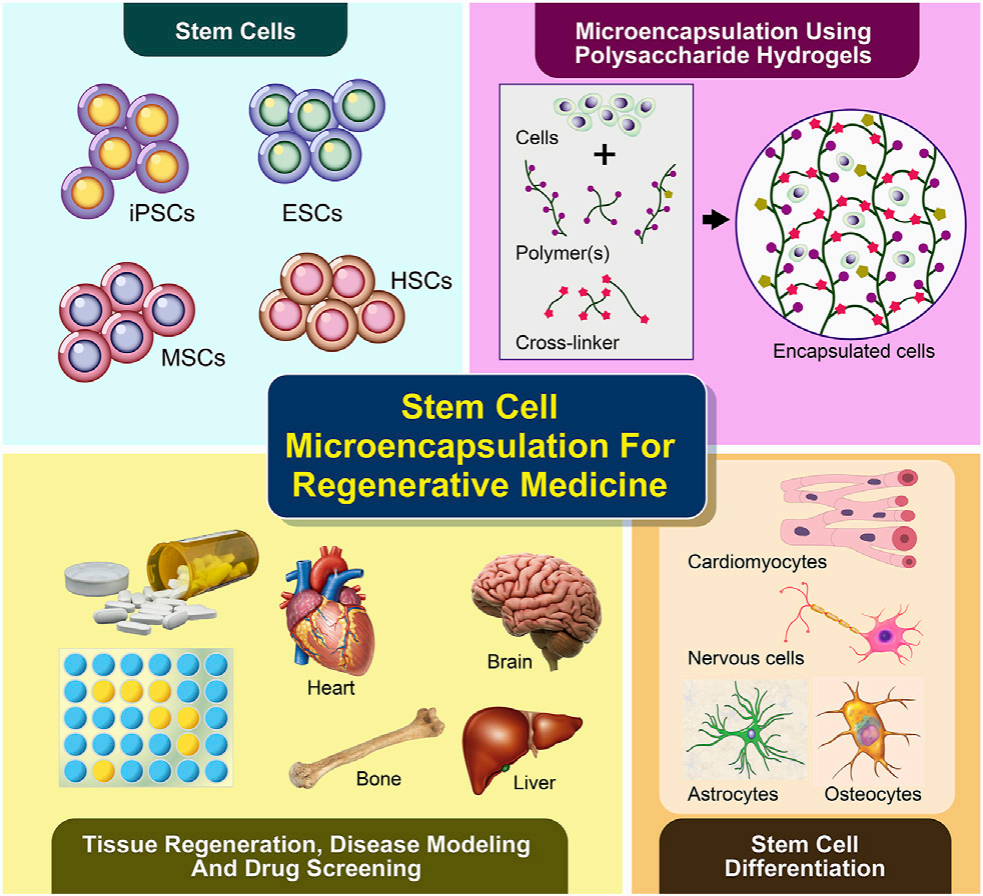
Microencapsulation of stem cells using polysaccharide-based hydrogels supports cell differentiation and viability in 3D, which has been recently applied in tissue regeneration or cell therapy and disease modeling for future drug screening.

Regenerative medicine offers great potential for restoring individual tissues or organs using patient’s stem cells incorporated with scaffolds (Mason and Dunnill, 2008). A number of stem cell-biomaterial related studies have been performed with the aim of treating various diseases and injuries, such as neurodegenerative disorders, diabetes, cardiovascular diseases, liver diseases, musculoskeletal defects, osteoarthritis and wound injuries (Crevensten et al., 2004; Kuo et al., 2008; Segers and Lee, 2008; Mazhari et al., 2020). Stem cells possess self-renewal capability and the potential to differentiate into multiple lineages, which include pluripotent stem cells (embryonic stem cells, ESCs and induced pluripotent stem cells, iPSCs) and multipotent stem cells (hemopoietic stem cells, HSC; mesenchymal stem cells, MSCs and adult stem cells, ASCs) (Leeper et al., 2010). Owing to their distinctive abilities and characteristics, stem cells have been identified as an unprecedented and important source of clinically relevant differentiated cells for application in regenerative medicine, particularly as cell delivery components for stem cell therapy and *in-vitro* (disease) models for drug discovery (Figure 1).

The use of biomaterial scaffolds, which can resemble intrinsic extracellular matrix (ECM) and direct stem cells, is crucial in the regeneration of functional tissues. It is challenging to design and develop scaffolds that can support cell survival, promote bioactivity and improve cell retention at the administered sites for cell delivery, cell transplantation as well as disease modeling (Parisi-Amon et al., 2013; Mayfield et al., 2014). In this respect, hydrogels are among the most promising biomaterials for recreating the native ECM properties due to their high-water content, biological compatibility and moldability (Slaughter et al., 2009). Various types of hydrogels made of natural polymers, synthetic polymers and co-polymers with optimized physical and chemical properties have been developed for regenerative medicine (Engler et al., 2006; Huebsch et al., 2010). Biophysical cues including porosity, degradation and mechanical strength or stiffness, have been incorporated into hydrogels in a spatiotemporally controlled manner to systematically modulate the behavior of stem cells such as cell proliferation, differentiation and migration (Yang et al., 2016). In addition, advanced chemical strategies and conjugation of functional materials or molecules were found to improve the cell-matrix interaction and functionality of hydrogels (Phelps et al., 2012). In the selection of hydrogel materials, natural polymers (e.g., polysaccharides and proteins) have gained much interest in the construction of ECM for stem cells and their derivatives owing to their hydrophilicity, biocompatibility, low cytotoxicity, biodegradability, softness, similarity to physiological environment and tunability into an injectable gel (Gomez-Florit et al., 2020).

This review focuses on the natural hydrogels derived from polysaccharides, including agarose, alginate, carrageenan, chitosan, gellan gum and hyaluronic acid. Despite major advantages of polysaccharide hydrogels, these materials are not without limitations. For example, they do not have strong mechanical properties, and some may not easily be controlled due to their batch-to-batch variation. For these reasons, polysaccharide hydrogels are often combined with protein-based or synthetic polymers, creating composite or co-polymer hydrogels, and are still widely experimented (Jabbari et al., 2016). In addition to aiding the retention of microencapsulated stem cells by providing biological and physical supports, polysaccharide hydrogels also serve as semi-permeable membranes with interconnected pores, which allows nutrient supply, mass transfer and waste removal from the microencapsulated cells. Hydrogels further protect the microencapsulated cells from immune attacks of host immune biosystems. They can be easily modified to incorporate various cell-interactive moieties to facilitate stem cell-based therapy by enhancing cell viability and specifically directing stem cell differentiation to target tissues. (Burdick and Vunjak-Novakovic, 2009; Guilak et al., 2009). Accordingly, this gives rise to the emergence of many methods for stem cell microencapsulation and application in regenerative medicine.

In the first part of this review, we discuss the type and characteristics of stem cells which have been widely used for microencapsulation and tissue regeneration. We also highlight selected polysaccharide polymers that can be processed under mild conditions to produce biomimetic hydrogels suitable for stem cell microencapsulation. The advanced microencapsulation techniques that allow the production of polysaccharide hydrogels with controlled size, in the form of beads, particles or capsules within the range of micrometers will be introduced. Finally, we further discuss the application of microencapsulated stem cells using biomimetic polysaccharide hydrogels in stem cell therapy or cell delivery and disease modeling.

## Stem Cells

Stem cells are unspecialized cells with the ability to self-renew and differentiate into at least one type of mature cells. Based on the potential of differentiation, stem cells can be classified into totipotent stem cells (able to generate all types of cells including germ cells), pluripotent stem cells (able to generate all types of cells except for cells of the embryonic membrane), and multipotent stem cells (able to generate more than one type of mature cells). In this section, we will discuss pluripotent stem cells (PSCs) e.g., ESCs and iPSCs, and two types of multipotent stem cells e.g., HSCs and MSCs.

### Embryonic Stem Cells

ESCs are derived from the inner cell mass (ICM) of the blastocyst, a pre-implanted embryo developed 4 days after fertilization. Isolation of the ICM can be achieved by immunosurgery or mechanical dissection*.* ESCs are cultivated by culturing with either feeder cells of various sources or cell-free media conditioned by fibroblasts and supplemented with appropriate growth factors. Notably, the three-dimensional (3D) culture system is preferred over the traditional two-dimensional (2D) culture system as it provides a niche that closely resembles the physiological environment. Multiple 3D cultures have been developed, with the most physiologically relevant matrix being hyaluronic acid (HA)-based hydrogel. Functions of human ESCs can be confirmed by their potential to differentiate into cells of all three germ layers *in vitro* as well as *in vivo* (teratoma formation assay in severe combined immunodeficiency mice). So far, many cell types have been successfully obtained from ESCs, including endoderm-derived hepatocytes, pancreatic beta cells, lung epithelium, mesoderm-derived bone, cartilage, cardiomyocytes, hematopoietic cells, endothelial cells, and ectoderm-derived keratinocytes, retinal pigment epithelium and neurons. The basic paradigm of PSC-based cell therapy is that PSCs are first differentiated into the desired cell type, followed by transplantation of the differentiated cells into patients (Vazin and Freed, 2010). While ESCs possess immense therapeutic potential, their use is limited by 1) ethical issues as human embryos are destroyed to isolate ESCs, and 2) transplanted cells derived from allogenic ESCs as they are subjective to host immune rejection. To this end, ESCs can be replaced by iPSCs (Moradi et al., 2019).

### Induced Pluripotent Stem Cells

The iPSCs are derived from somatic cells that are dedifferentiated *in vitro* to a pluripotent stage using either an integrative or non-integrative approach. In the former, retroviral or lentiviral vectors are used to deliver four reprograming factors (Oct4, Sox2, Klf4, and c-Myc). The latter approach involves episomal DNA plasmids, Sendai virus, adenovirus, synthesized modified mRNA, microRNAs, proteins and small molecules. Compared to the integrative approach, the non-integrative approach has a lower reprograming efficiency but a minimal risk of inducing mutagenesis, and is therefore considered more suitable for stem cell-based therapies (Moradi et al., 2019). Like ECSs, the growth of iPSCs *in vitro* also requires appropriate extracellular matrices and environmental cues, which can be achieved with the introduction of animal cells, hydrogels, individual matrix proteins, synthetic surfaces, and some commercially well-defined and xenogeneic-free components (Chen et al., 2014). However, directing iPSCs to a specific cell lineage remains a challenge and each differentiation progress likely requires unique features in the culture system. While a 3D culture system is favorable to the generation of many other cell types, a recent study has shown that it may impair the differentiation of iPSCs towards mesenchymal stem cells-like phenotype as compared to a 2D culture system (Goetzke et al., 2019). iPSCs are equally suitable for all the biomedical applications of ESCs, such as drug screening, toxicological studies, disease modeling and cell therapies. Several iPSC-based clinical trials to treat macular degeneration, Parkinson’s disease and heart diseases are ongoing. In recent cases where iPSCs are derived from a patient with certain disease-causing genetic mutations, cell therapy can potentially revert the mutations by applying CRISPR/Cas9 technology (Moradi et al., 2019).

### Mesenchymal Stem Cells

MSCs are commonly derived from adult human bone marrow and adipose tissue stromal vascular fraction. MSCs can be genetically distinguished from non-MSCs with an “MSC classifier” based on their gene expression profile. The number of MSCs in bone marrow is low but a large number of cells can be acquired by *in vitro* expansion. MSCs can be differentiated into various types of mesodermal tissues, including cartilage, bone, adipose tissue, stroma, muscle and tendon. This process requires treatment of MSCs with specific stimuli introduced in specific chronological order (temporal stochasticity). Differentiation of MSCs *in vitro* is also affected by the cellular environment (e.g., hypoxia, inflammatory cues) and the properties of the substrate. For example, rigid culture surfaces have been shown to favor osteogenesis whereas soft gels are conducive to adipogenesis. Due to the ease of isolation and expansion, MSCs have been widely applied in regenerative medicine. Over the past decade, however, the focus of MSC application has shifted from cell replacement to the paracrine function of MSCs. MSCs have been found to secrete multiple growth factors, cytokines, and immunomodulatory molecules, which is a unique feature of MSCs among the other stem cell types (Pittenger et al., 2019). In order to scale up the production of MSCs for clinical applications, 3D culture systems such as microcarriers and stirred-tank bioreactors, are the most common to achieve a higher surface-to-volume ratio than monolayer cultures (Koh et al., 2020; Tsai et al., 2020).

### Hemopoietic Stem Cells

HSCs are traditionally harvested from bone marrow but now predominantly from cytokine-mobilized peripheral blood stem cells. CD34 ^+^ peripheral blood stem cells are enriched using immunomagnetic separation and characterized by flow cytometry based on the expression of specific cell markers (CD34^+^, CD38^−^, CD45RA^−^, CD90^+^, CD49f) (Morgan et al., 2017). HSCs are able to produce all types of mature blood cells through differentiation of increasingly lineage-specific progenitors, which is regulated by both intrinsic factors (transcription factor, epigenetic regulators, and metabolic pathways) and extrinsic factors (humeral and neural signals, and local microenvironmental cues) (Pinho and Frenette, 2019). Bone marrow transplantation has been a curative therapy for a variety of hematological diseases over the last 4 decades and its implication has been advanced with gene editing techniques. However, bone marrow transplantation is still hindered by the immunological complications of allogenic transplantation as well as the suboptimal *ex vivo* expansion of HSCs in monolayer culture to provide sufficient amount of stem cells for marrow reconstitution (Ng and Alexander, 2017; Pinho and Frenette, 2019). 3D cultures may improve HSC yields by providing space as well as a more faithful simulation of tissue microenvironment than 2D. Recently, a 3D scaffold made of polydimethylsiloxane to mimic the natural hematopoietic niche has been demonstrated to support the viability, multipotency and self-renewal of human HSCs *in vitro* (Marx-Blümel et al., 2020)*.*

## Polysaccharide Hydrogels

### Agarose

Agarose is extracted from red algae and seaweed and consists of a galactose-based backbone 1,4-linked 3,6-anhydro-α-1-galactose and 1,3-linked β-D-galactose derivatives (Zarrintaj et al., 2018). It has a thermoresponsive property, i.e., in a gel state at room temperature but in a solution state at an increased temperature. This makes agarose a favorable biomaterial for its easy tunable mechanical properties during synthesis. Agarose solutions containing cells can be prepared and emulsified at 37°C, then gelated to microgels in an ice bath. As shown in Table 1, previous and current studies of stem cell encapsulation in agarose have been reported. An earlier research activity showed that agarose was used as a scaffold for vascular endothelial growth factor (VEGF) immobilization to encapsulate and differentiate ESCs during early stages of development toward blood progenitor cells (Rahman et al., 2010). Stem cells were encapsulated into agarose microwells to form structures known as ‘lockyballs’. The ‘lockyball’ interior structure consisted of an aggregate of human adipose stem cells that is surrounded by a synthetic coating, which contained multiple binding sites for other ‘lockyballs’ (Silva et al., 2016). In addition, agarose has been used as a printable bioink for generating specific tissues from human stem cells where recent work illustrated how MSCs are printed using an agarose-based bioink at different formulations, which can provide a versatile platform for stem cell therapy (Forget et al., 2017).

**TABLE 1:** Polysaccharides derived natural hydrogels microencapsulated with different type of stem cells and their response.

Polysaccharide material	Gelation mechanism	Stem cell type^1^	Significant biological responses	References
Agarose	Thermal	ESCs	Agarose hydrogel functionalized with VEGFA and successfully induced blood progenitor cells	Rahman et al. (2010)
		ASCs	Bio-fabricated ASCs spheroids into ‘lockyballs’ enabled spheroid aggregation, delivery and engraftment	Silva et al. (2016)
		MSCs	3D bioprinting agarose hydrogel supported MSCs survival in a tissue-like structure composed of a range of mechanically discrete microdomains	Forget et al. (2017)
Alginate	Ionic/chemical crosslinking	ESCs	Co-encapsulated functional β-like cells from human ESCs and CXCL12 enhanced insulin secretion in diabetic mice whilst evaded the pericapsular fibrotic response	Alagpulinsa et al. (2019)
		iPSCs	Alginate hydrogel functionalized with RGD peptide supported survival of functional neurons and allowed optogenetic stimulation	Lee et al. (2019)
		MSCs	Microfluidics encapsulated single-cell MSCs in alginate microgels enhanced osteogenesis and accelerated mineralization	An et al. (2020)
Carrageenan (CRG)	Thermal Ionic crosslinking	ASCs	Injectable k-CRG and non-injectable CRG co-encapsulated with TGF-β1 increased cell viability, induced chondrogenic differentiation and expression, and synthesized proteoglycans	Popa et al. (2015)
		MSCs	Excellent structural strength and optimal concentrations obtained by 3D bioprinted CRG-alginate composite without significant negative effects on the cell viability	Kim et al. (2019)
		iPSCs and MSCs	Micropatterned κ-CRG hydrogel systems of defined shapes supported the growth of stem cells and enabled the spatial control of stem cell niche	Vignesh et al. (2018)
Chitosan	Ionic/chemical crosslinking	ESCs	Chitosan incorporated with VEGF and endothelial cells to induce neovascularization	Lee et al. (2015)
		MSCs	Injectable thermo-responsive chitosan promoted wound healing, supported MSC’s secretion of growth factor and inhibited inflammation factors	Xu et al. (2019b)
		ASCs	N-methacrylate glycol chitosan (MGC) hydrogels incorporated with RGD peptide sustained cell viability, increased cell spreading and metabolic activity. Encapsulated ASCs promoted murine CD31^+^ endothelial cell recruitment to the peri-implant region	Dhillon et al. (2019)
Gellan Gum (GG)	Thermal Ionic/chemical crosslinking	ESCs and iPSCs	Bioamine-crosslinked and laminin-functionalized GG hydrogel further induced neural cell viability, maturity and supported neurite migration	Koivisto et al. (2017)
		ASCs	GG composited with collagen type-1 and bioactive glass was found to support osteogenic differentiation of ASCs	Vuornos et al. (2020)
		iPSCs	The covalent hydrazone crosslinking GG blended with gelatin supported a prolonged culture of cardiomyocytes in 3D, allowed the cardiac model to maintain its elasticity and closely mimicked the native heart for at least 7 days	Koivisto et al. (2019)
Hyaluronic acid (HA)	Thermal Ionic/chemical crosslinking	iPSCs	The soft 3D methacrylated hyaluronic acid (Me-HA) hydrogel-encapsulated hiPSC-NPCs displayed robust neurite outgrowth and showed high level of spontaneous neural differentiation	Wu et al. (2017)
		MSCs	Encapsulation of human vascular endothelial-cadherin (hVE-cad-Fc) fusion protein functionalized MSC aggregates (FMA) using HA-based hydrogel demonstrated better recovery of cardiac function and improved revascularization of infarcted myocardium in comparison to the conventional hydrogel-MSC delivery system	Lyu et al. (2020)
		ESCs	HA backbone was chemically modified with gelatin to encapsulate and deliver hESC-neural stem cells, successfully improved locomotor function in a rat spinal cord injury model	Zarei-Kheirabadi et al. (2020)

1 ESCs: Embryonic Stem Cells; iPSCs: induced Pluripotent Stem Cells; MSCs: Mesenchymal Stem Cells; ASCs: Adipose-derived Adult Stem Cells.

### Alginate

**FIGURE 2:**
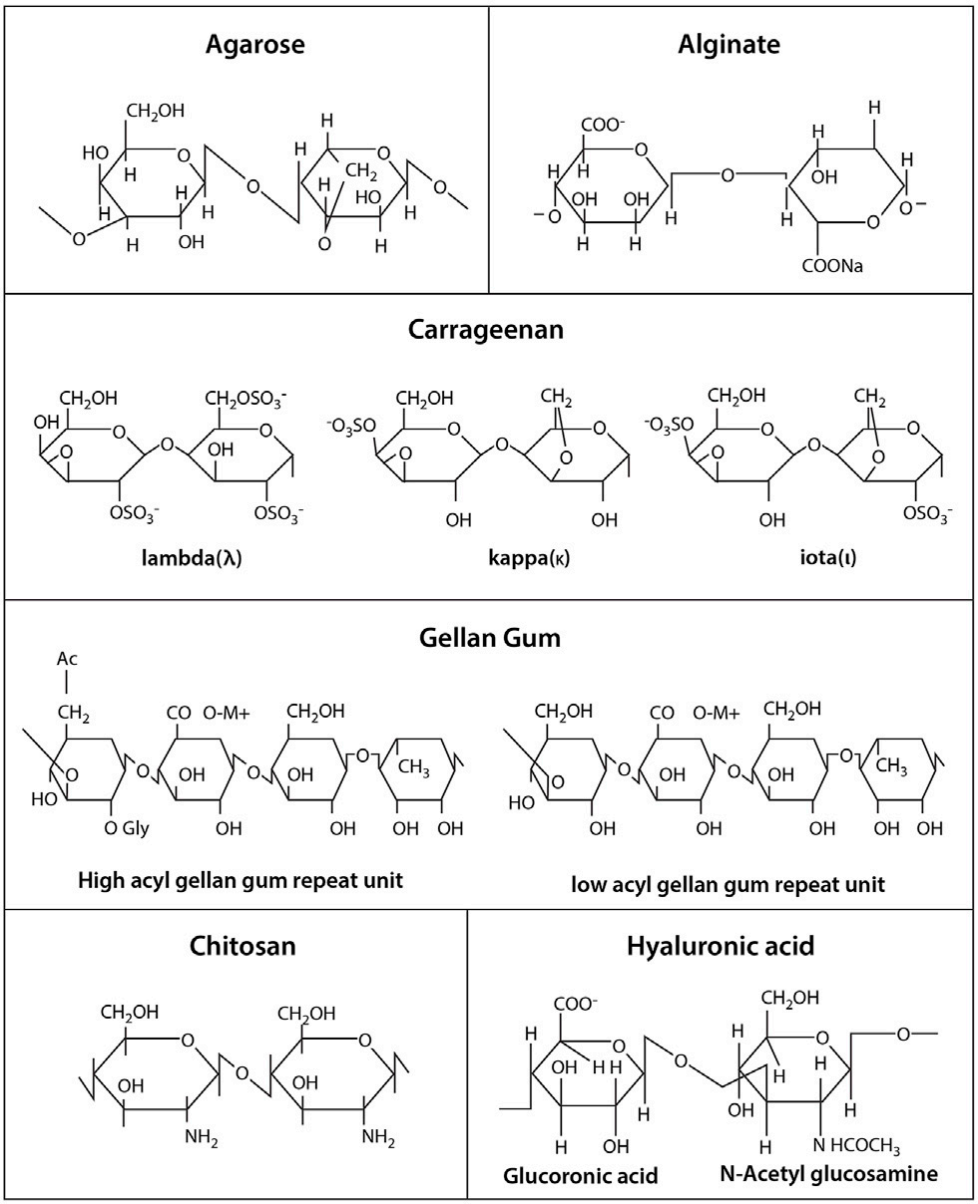
Chemical structures of polysaccharide hydrogels.

Alginate is a polysaccharide derived from brown algae containing guluronic acid (G units) and mannuronic acid (M units) (Figure 2). Alginate-based biomaterials have been widely used for biomedical and pharmaceutical applications because of their biocompatibility and ionic crosslinking. It has been the most popular natural polymer matrix for cell microencapsulation due to quick gelation without using toxic chemicals or organic solvent (Andersen et al., 2015; Ching et al., 2017). Divalent cations such as calcium, barium or magnesium are used as ionic crosslinkers to form ionic bridges between alginate G units. The most frequent used crosslinker, calcium chloride (CaCl_2_), is highly soluble in aqueous medium which can trigger rapid or poorly controlled gelation. To decrease the gelation rate, calcium carbonate (CaCO_3_) or calcium sulfate (CaSO_4_) is added. For example, calcium ions (Ca^2+^) will be released from CaCO_3_ when glucono-δ-lactone is applied in alginate/CaCO_3_ mixture, subsequently initiating alginate gelation in a gradual manner (Crow and Nelson, 2006). Furthermore, the Ca^2+^ release from alginate hydrogel may induce hemostasis, which leads to interest in covalently crosslinking.

In stem cell encapsulation, alginate hydrogel was combined with ESCs to generate functional human beta-like cells (SC-β cells) (Maguire et al., 2006). The capacity of these cells to co-microencapsulate with immunomodulatory chemokine (CXCL12) in alginate can evade the fibrotic foreign body reaction and induce long-term glycemic correction in an immunocompetent murine model of type-1 diabetes without systemic immunosuppression (Maguire et al., 2006; Alagpulinsa et al., 2019). Bone and cartilage have also been regenerated using microfluidics or bioprinting methods where MSCs or iPSCs were not only encapsulated in suspension. However, single-cell encapsulation has been achieved lately to prevent non-homogeneous differentiation (Nguyen et al., 2017; An et al., 2020).

Although alginate has been fundamentally easy to utilize for stem cell microencapsulation, it lacks biological active moieties. In order to improve cell-cell and cell-matrix interaction for efficient stem cell-based therapy, alginate has been modified, coated or composited with other biologically active molecules or polymers (e.g., gelatin, hyaluronic acid, chitosan, poly-L-lysine, various growth factors, peptides and proteins). For instance, when a combination of alginate and HA hydrogel was formulated for MSC microencapsulation, an optimal composition of 1% alginate and 0.25% HA was found to greatly enhance cell growth and support release of therapeutic proteins (Cañibano-Hernández et al., 2017). Lee and co-workers covalently conjugated alginate with ECM-derived peptide (e.g., arginine-glycine-aspartic acid, RGD),successfully stimulated iPSCs and neural derivatives, promoted cell viability and differentiation as well as allowed optogenetics application in the 3D culture system (Lee et al., 2019). Hence, alginate-based hydrogels can be further tailored to better resemble the natural ECM microenvironments by providing multiple specific signals to stem cells and their derivatives.

### Carrageenan

Carrageenan is a sulphated polysaccharide extracted from red seaweeds (Rhodophyceae), which contains repeating disaccharide units of 4-linked b-D-galactopyranose (G-unit) and 4-linked a-D-galactopyranose (D-unit) or 4-linked 3,6-anhydrogalactose (DA-unit), with a variable portion of sulphate group (Campo et al., 2009). It can be categorized into three main families - kappa (κ), iota (ι) and lambda (λ), based on the number and position of the sulphate group in the repeating galactose units. Among them, κ-carrageenan (k-CRG) has primarily and recently been exploited in cell therapy due to its distinguishing properties, including thermoresponsive nature, facile gelation, moldability, close resemblance to glycosaminoglycans (GAGs) and good injectability under physiological conditions (Campo et al., 2009; Mohite and Patil, 2014). Stem cells are encapsulated within κ-CRG hydrogels in a mild condition with ionic gelation mechanism (Mohite and Patil, 2014).

Several recent studies show a promising performance of κ-CRG hydrogels. For example, injectable κ-CRG hydrogels encapsulated with human ASCs and TGF-β1 for cartilage regeneration were reported to enhance cell viability and proliferation, increase chondrogenic differentiation and expression level, and stimulate production of proteoglycans and other ECM components of cartilage (Popa et al., 2015). Carrageenan has also been compositely incorporated with other polymers such as alginate and chitosan for preparing hydrogel beads and fibers. It has demonstrated good processability at different formulations for application in tissue regeneration and cell delivery (Kim et al., 2019). Recently, a bioprinted co-polymer hydrogel consisting of carrageenan and alginate encapsulated with MSCs, has demonstrated excellent structural strength and biological activity (Kim et al., 2019).

### Chitosan

Another commonly used polysaccharide polymer, chitosan, is derived by the deacetylation of chitin. It consists of glucosamine units such as β-(1→4)-linked D-glucosamine and N-acetyl-D-glucosamine (Wan and Tai, 2013). Chitosan has been extensively used in tissue regeneration because of its excellent biocompatibility, biodegradability, hydrophilicity and structural similarity to glycosaminoglycans (GAGs) (Kim et al., 2008). The gelation of chitosan hydrogels can be controlled using pH (Chang et al., 2015) and the hydrogel properties can be modified for stem cell encapsulation through chemical crosslinking (Muzzarelli et al., 2016). Numerous works showed that 3D chitosan hydrogels at different concentrations promoted osteogenic and chondrogenic differentiation of human stem cells (Muzzarelli et al., 2015). Other researchers have combined chitosan hydrogels with stem cells and growth factors to treat spinal cord injury (Li et al., 2016). Chitosan-based hydrogels have been further modified or functionalized to increase the biological activities of encapsulated cells (Dhillon et al., 2019). Since it can also provide analgesic effect and hemostatic activity, much current research focuses on its application in wound healing (Xu H. et al., 2019; Soriano-Ruiz et al., 2019). For example, injectable thermo-sensitive hydrogel loaded MSCs from umbilical cord blood was found to successfully accelerate wound closure and support tissue remodeling and regeneration of skin appendages for cutaneous wound healing. (Xu Y. et al., 2019; Soriano-Ruiz et al., 2019). Furthermore, chitosan-based hydrogel encapsulating ESC-derived endothelial cells and VEGF induced robust cell retention and promoted neovascularization through vasculogenesis and angiogenesis (Lee et al., 2015). Recently, studies show chitosan bioink is suitable for printing stem cell-derived constructs (Roehm and Madihally, 2018). However, optimization and more studies are required to ensure stem cell survival and differentiation.

### Gellan Gum

Gellan gum (GG), an anionic polysaccharide polymer, is obtained from *Sphingomonas Elodea*. It contains repeating units of β-D-glucose, β-D-glucuronic acid and α-L-rhamnose in a molar ratio of 2:1:1 (Prajapati et al., 2013). It has been immensely used for the encapsulation of drugs, enzymes, cells and microorganism attributed to its hydrophilicity and excellent gelling property in the presence of cations (Wang et al., 2008; Chakraborty et al., 2014).

Like many other polysaccharide polymers, GG is a relatively inert biomaterial that requires further modification and improvement to support cell adherence. GG-based hydrogels are functionalized with various type of peptides by covalently conjugating them into the molecular backbone itself (Chakraborty et al., 2014). GG has been studied for the regeneration of bone (Vuornos et al., 2020), cartilage (Park et al., 2020) and spinal cord (Gomes et al., 2016). In neural regeneration, Koivisto and co-workers have demonstrated that bioamine-crosslinked GG hydrogels supported viability of both ESCs and iPSCs derived neuronal cells, and further confirmed that functionalized GG hydrogels with laminin resulted in cell type-specific behavior, neuronal cell maturity and neurite migration (Koivisto et al., 2017). Other studies reported that the development of composite GG, incorporated with collagen type-1 and bioactive glass, can support the osteogenic induction of human ASCs (Vuornos et al., 2020). This suggested that a specific type of peptide/protein, growth factor and composite material plays a key role in triggering specific stem cell differentiation, hence these factors need to be considered in hydrogel synthesis and modification.

### Hyaluronic Acid

Hyaluronic acid (HA), also known as hyaluronan, is one of the major components of ECM and consists of multiple sites for cell adhesion (Knopf-Marques et al., 2016). It is a non-sulphated glycosaminoglycan with repeating disaccharide units of glucoronate and N-acetylglucosamine (Khademhosseini et al., 2006). Many studies have demonstrated that HA regulates stem cell niches, thus making it suitable for stem cell microencapsulation and culture (López-Ruiz et al., 2019). HA has been developed as a hydrogel scaffold for promoting self-renewal and vascular differentiation of human ESCs (Gerecht et al., 2007). Other research groups detailed the incorporation of bone marrow derived MSCs with injectable HA hydrogel to engineer cartilage tissue (Jooybar et al., 2019), and MSCs encapsulated with a photocrosslinkable HA-collagen hydrogel to generate bone tissue (Zhang et al., 2019). Tissue regeneration using encapsulated stem cells in HA appears as a promising strategy to promote wound healing as well as to repair damaged nerve tissues (da Silva et al., 2017; Wu et al., 2017). In addition, HA microcarriers and HA bioinks provide a conducive environment for stem cell growth (Shendi et al., 2017; Law et al., 2018; Sakai et al., 2018). For instance, HA blended with methylcellulose supported MSCs viability at above 75% in the bioprinted structures, and the cells retained viability for at least 1 week after 3D bioprinting (Law et al., 2018).

## Microencapsulation Techniques

### Extrusion

**FIGURE 3:**
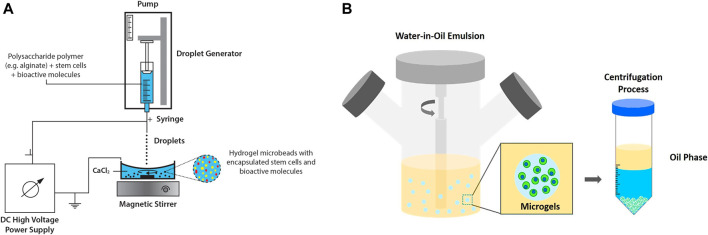
Extrusion and emulsion methods. **(A**) Electrostatic/electrospray droplet extrusion technique for microencapsulation of stem cells and bioactive molecules. Figure adapted with permission from Figure 1 of (Verica et al., 2008). **(B)** Emulsification technique (water-in-oil emulsion) produces hydrogels for cell encapsulation. Figure adapted with permission from Figure 6 of (Choe et al., 2018).

Extrusion method, which includes air jet extrusion, syringe droplet extrusion, centrifugational extrusion, electrostatic extrusion and vibrational extrusion, is among the most common methods applied to microencapsulate cells for regenerative medicine. Among the extrusion methods, electrostatic/electrospray droplet extrusion has been widely applied for stem cell microencapsulation study (Kim et al., 2019) Hydrogel beads of approximately 50 µm are produced and the reduction of cell viability can be avoided by the use of organic solvent. The process involves gravitational dripping where a suspension of hydrogel precursor and cells are extruded via a needle into a hardening solution (Figure 3A) (Hashemi and Kalalinia, 2015).

There are several factors that influence the diameter of the cell encapsulated microspheres such as density of solution, diameter of the extrusion needle/nozzle and surface tension of the droplets. Peng and co-workers reported the optimization of electrospray technique to encapsulate human bone marrow stromal cell (hBMCs) in alginate-gelatin microspheres (Xu H. et al., 2019). In the study, non-aggregated, polydispersity and defined spherical microspheres were produced with alginate (1.5%, w/v) and gelatin (0.5%, w/v) using a 30 G needle and 8 kV voltage. When compared to alginate microsphere alone, alginate-gelatin improved cell proliferation and viability by up to 21 days. A method to control cell-release tunable microbead hydrogels containing adipose stem cells (ASCs) had also been developed (Leslie et al., 2017). In this study, electrostatic extrusion dripping was employed to produce enzymatically modulated hydrogels. Nevertheless, a major limitation of this technique is the presence of cells which often leads to the clogging of nozzle. In certain cases, nozzle inner diameter and applied pressure are two factors that cause cell damage (Cidonio et al., 2019). The clogging issue can be reduced by ensuring cell suspension is homogenous as well as flushing the nozzle with sodium citrate. Meanwhile, employing right parameters and a blunt nozzle may prevent cell damage.

### Emulsion

Cell encapsulation by emulsion method is typically carried out by dispersing hydrogel precursor in non-miscible solution, namely water-in-oil emulsion. At equilibrium, internal gelation occurs in which the emulsified hydrogels are later collected by a centrifugation process (Choe et al., 2018; Figure 3B). Despite advantages of this technique e.g., low production cost and high scalability, broad size distribution and cell disruptions at oil interface have raised some concerns (Daly et al., 2020). Prolonged exposure towards oil and surfactants resulted in cytotoxic environment that disrupted cells and subsequently reduced cell viability (Chan et al., 2013). Water-in-water emulsion drop, which involved a one-phase system, has been reported as a template to produce microgels. However, a specific combination of two immiscible aqueous solutes is required, which would limit the potential use of this modified approach. To obtain uniform micro encapsules, a few studies have investigated the application of double emulsion technique (Chan et al., 2013; Liu E. Y. et al., 2018). Choi and co-workers adopted double emulsion drop technique with ultrathin oil shell being as a sacrificial template (Choi et al., 2016) The monodisperse emulsion drops were formed via coaxial flow aqueous pre-polymer solution surrounded by oil phase and directly emulsified into a continuous aqueous phase. Upon UV exposure, dewetting occurred and the hydrogels solidified. The researchers demonstrated that this approach can support large-scale hydrogel production and increase cell viability attributed to the shorter exposure of cells to oil phase (Choi et al., 2016).

### Lithography

**FIGURE 4:**
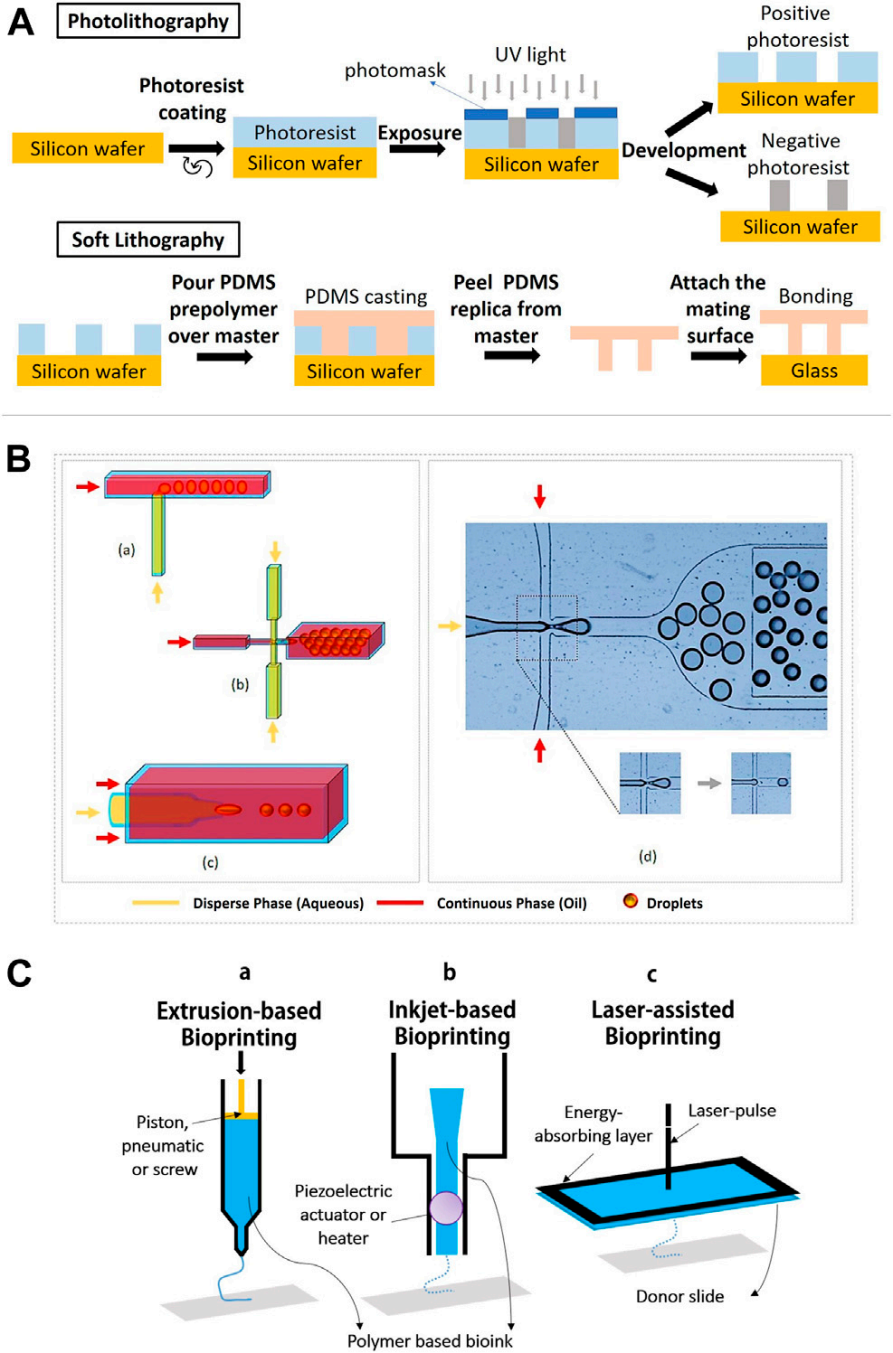
Lithography, microfluidics and bioprinting methods for stem cell microencapsulation. **(A)** Schematic diagram shows the process of photolithography and soft lithography. **(B)** Geometric illustrations of three microfluidic devices: (a) T-junction, the perpendicular flow of the continuous phase is sheared by dispersed phase and thereby generates droplets. (b) Flow-focusing, droplets are produced by shearing the dispersed phase from two directions. (c) Co-flow, the dispersed phase is forced through a capillary inside a bigger capillary where continuous phase is pumped. (d) Droplet generation by flow-focusing device (use of fluorinated oil with stabilizing agent and dispersed phase as water solution of dye). Figure adapted with permission from Figure 1 of (Hayat et al., 2018; Alkayyali et al., 2019). **(C)** Schematic diagram of (a) piston extrusion-based bioprinting, (b) piezoelectric actuator inkjet-based bioprinting, and (c) laser-assisted bioprinting.

There are two common methods of lithography for the fabrication of microfluidic cell culture devices, namely photolithography and soft lithography. This technique is used to pattern hydrogels at the micro and nanoscale with bioactive features to improve cell differentiation, spreading and migration (Guan et al., 2017) In photolithography, a silicon wafer is spin-coated with a viscous photoresist, which will start to crosslink when exposed to high energy of UV light. The designed pattern of hydrogel is formed (Figure 4A). Soft lithography has been introduced to replicate a mold of the microstructure, allowing nanofabrication by pouring a polymer solution or spin-coated onto a master for crosslink until a rubbery replica is formed (Gasperini et al., 2014) The channels in the replica can be filled or loaded with a suspension of a hydrogel precursor and cells. Master is a photoresist patterned silicon wafer with polydimethylsiloxane (PDMS) chosen as an elastomer to cover the surface of the master because of its transparency, gas permeability and biocompatibility (Tang et al., 2021). In addition, soft lithography possesses unique advantages as it could provide a good resolution (∼35 nm), which is more competitive when compared to electron beam lithography (∼15 nm) (Lin et al., 2018). By using soft lithography, the fabrication of polymer materials allows procedures to pattern non-planar substrates with a wide range of materials (Rose et al., 2019).

Currently, several advanced lithography techniques have been established such as microcontact printing, replica molding, micro-transfer molding and solvent assisted micro-molding/micropatterning. Previous research by Suh and co-workers demonstrated that a soft lithographic technique using HA is compatible with microcontact printing and molding approaches (Suh et al., 2004). During microcontact printing, PDMS stamps were used with oxygen plasma in order to enhance the adhesion of HA to the stamps. Results showed that the pattern transfer by this method had a good edge definition where the height of the printed HA was higher (∼90 nm) than typically obtained by self-assembled monolayers. However, microcontact printing and photolithography are restricted to many other polymers which require ionic or thermal crosslinking. Series of fabrication steps are laborious and often damage encapsulated cells during curing and demolding. Recent work to overcome these issues by using simple paraffin wax molds was reported to successfully generate defined shapes on alginate-gelatin and κ-carrageenan hydrogel surface. This supports the viability of both MSCs and iPSCs (Vignesh et al., 2018).

Moreover, an advanced lithography based 3D bio-printing has also been introduced where both UV and visible light can be applied to cure photocrosslinkable bioinks as well as to improve the resolution and to achieve multi-material printing ability (Liang et al., 2020). Factors such as temperature change, curing or drying during processing, UV initiators in UV crosslinking and light intensity could have detrimental effects on the viability of encapsulated stem cells. Thus, further improvement and optimization are required.

### Microfluidics

Microfluidics is a method which deals with the handling of fluids in microenvironments that allows the formation of micro gels. In stem cell culture, microfluidics involves a small-scale system, which focuses on the flow volume and channel size, and is increasingly being explored (McKee and Chaudhry, 2017). The microfluidics is also used to simulate the *in vivo* microenvironment via perfusion media exchange and creating chemical gradients of soluble factors for low amount of cells or single cells (Halldorsson et al., 2015). Droplet-based microfluidics appears as a powerful method and versatile technique to reconstruct microenvironments with remarkably high throughput and tight control over cells, biomolecules and extracellular matrix. There are active and passive methods of droplet-based microfluidics. The most common devices in cell microencapsulation are derived from passive methods, including flow-focusing, co-flow and T-junction design (Figure 4B) (Rossow et al., 2017). Details on the active and passive droplet-based microfluidics methods can refer to the recent review article (Zhu and Wang, 2017). Generally, flow-focusing and co-flow microfluidic devices form droplets as a reaction to shear stress of a continuous phase on a dispersed phase. Both phases are normally immiscible liquids. Meanwhile for the T-junction devices, a droplet is created when the two channels collide with each other at the right angle and leave through a perpendicular stem (Alkayyali et al., 2019). The size and shape of the droplets in microfluidics-based synthesis is influenced by the sizes of the microchannel and flow rates of the two phases. T-junction is a common technique in microfluidics for cell microencapsulation owing to ease of droplets and uniform size distribution of microbeads (Alkayyali et al., 2019). Stem cells such as ESCs, have been embedded in agarose hydrogel using T-junction technique (Kumachev et al., 2011).

### Bioprinting

Bioprinting is an emerging technology for tissue regeneration because of its ability to produce bio-artificial organs and to mimic the cell-matrix native microenvironments (Lei and Wang, 2016). In general, 3D bioprinting is utilized to deposit biomaterials layer by layer with the assistance of digital 3D computer aided design (CAD). There are three common techniques employed in advanced tissue regeneration and organ manufacturing areas. They are inkjet bioprinting, laser-assisted bioprinting and extrusion-based bioprinting (Figure 4C). The working principle in the inkjet bioprinting technology is simple and similar to home printing techniques where the hydrogel is printed separately layer by layer using thermal and acoustic methods. The heat from the printer head forces the cells and biomaterials out of the nozzle through pressure pulses. In addition, extrusion-based bioprinting uses the same extrusion principle where the fluids are released by a pressure assisted system. In laser-assisted bioprinting, a vapor pressure (laser pulse) forms bubbles between the solution and a piece of glass (donor slide) where the pressure will shoot a small droplet of solution towards the collector substrate drop by drop. Subsequently, the repeated processes produce a tissue-like structure. During the process, the polymer solution is transformed into a 3D structure by crosslinking, which involves ionic crosslink or UV photo polymerization (Markstedt et al., 2015). The printing temperature is set between 1°C and 37°C to avoid causing overheating damage to the encapsulated cells (Lei and Wang, 2016).

The important feature of 3D bio-printers is to print living cells together with polymeric hydrogels and other bioactive compounds, either alone or in combination with other polymers as the main composition of bioinks. This will impact the mechanical and cellular behaviors of the generated biological structures. Previous studies showed that the use of polysaccharide polymers and copolymers as bioinks can produce a stable microenvironment for stem cells to grow, proliferate, differentiate and migrate (Liu F. et al., 2018). There are four types of hydrogel bioinks that are classified based on the crosslinking methods such as ionic-crosslinked bioink, thermo-sensitive bioink, photosensitive bioink and shear-thinning bioink (Xu et al., 2020). Each bioprinting technique has limitations and different requirements for the bioinks which can affect the encapsulated stem cells. Although inkjet bioprinting and laser-assisted bioprinting are able to position multiple cell types accurately with high cell survivability, inkjet bioprinting has the limitations of vertical printing and restricted material viscosities to produce a 3D architecture, whereas laser bioprinting only positions the bioink onto a prefabricated matrix and suffers from less stability, low scalability and high cost. In contrast, extrusion bioprinting has quick fabrication times for any 3D microstructures but poor cell viability. Thus, combining different bioprinting techniques could solve the existing limitations and adopt advantages from each other (Željka et al., 2018). Current research demonstrates the feasibility and efficiency of using more than one cell microencapsulation technique. Integrating 3D bioprinting (digital micromirror device (DMD)-based projection printing) and microfluidics improved bioprinting time and speed with less than 20 s for two to three bioinks and allowed more than one type of cell (Amir et al., 2018).

### Superhydrophobic Surfaces

Hydrophilicity of most pristine hydrogels can cause inert characteristics and affect the functionality of the hydrogels. In an encapsulated hydrogel system, small molecules and solutes can freely diffuse across the hydrogel layer. Nevertheless, in certain cases where larger molecules or components present in the system, this may block the interaction of the cells and the hydrogel matrix (Pérez-Luna and González-Reynoso, 2018). It has been shown that surface-coating hydrogels with a super hydrophobic surface can prevent contamination within hydrogel beads and control the entrance of solvents for molecular exchange with the surrounding environment (Lima et al., 2011). This technique was adopted to produce alginate hydrogels coated with polystyrene surfaces and crosslinked with CaCl_2_ to encapsulate MSCs and fibronectin (Lima et al., 2013). It is practically important to entrap water soluble molecules such as fibronectin, which can easily diffuse to the media. In the study, MSCs isolated from Wistar rat’s bone marrow were immobilized into alginate beads through a process of gelification of liquid precursor droplets onto biomimetic superhydrophobic surfaces. In the microencapsulation and gelification process, no additional process of precipitation and aggressive mechanical strength were used, the hydrogel was rapidly formed without aggregations in 5 min. The results demonstrated that alginate beads at 2% of concentration were found to remain stable for 21 days whilst hybrid bone regeneration was accelerated. Major advantages of this technique, including mild processing conditions, controllable hydrogel size, high encapsulation loading, and mechanical forces are not required during formation of the particles. However, it is difficult to bind and incorporate with other superhydrophobic nanoparticles without sacrificing or degrading its superhydrophobic nature. Several in-depth reviews are available, which focus on the materials and methods used to produce superhydrophobic surfaces (Eric et al., 2016; Karim et al., 2019).

## Moving Towards Clinical Translation

### Stem Cell Therapy

**FIGURE 5:**
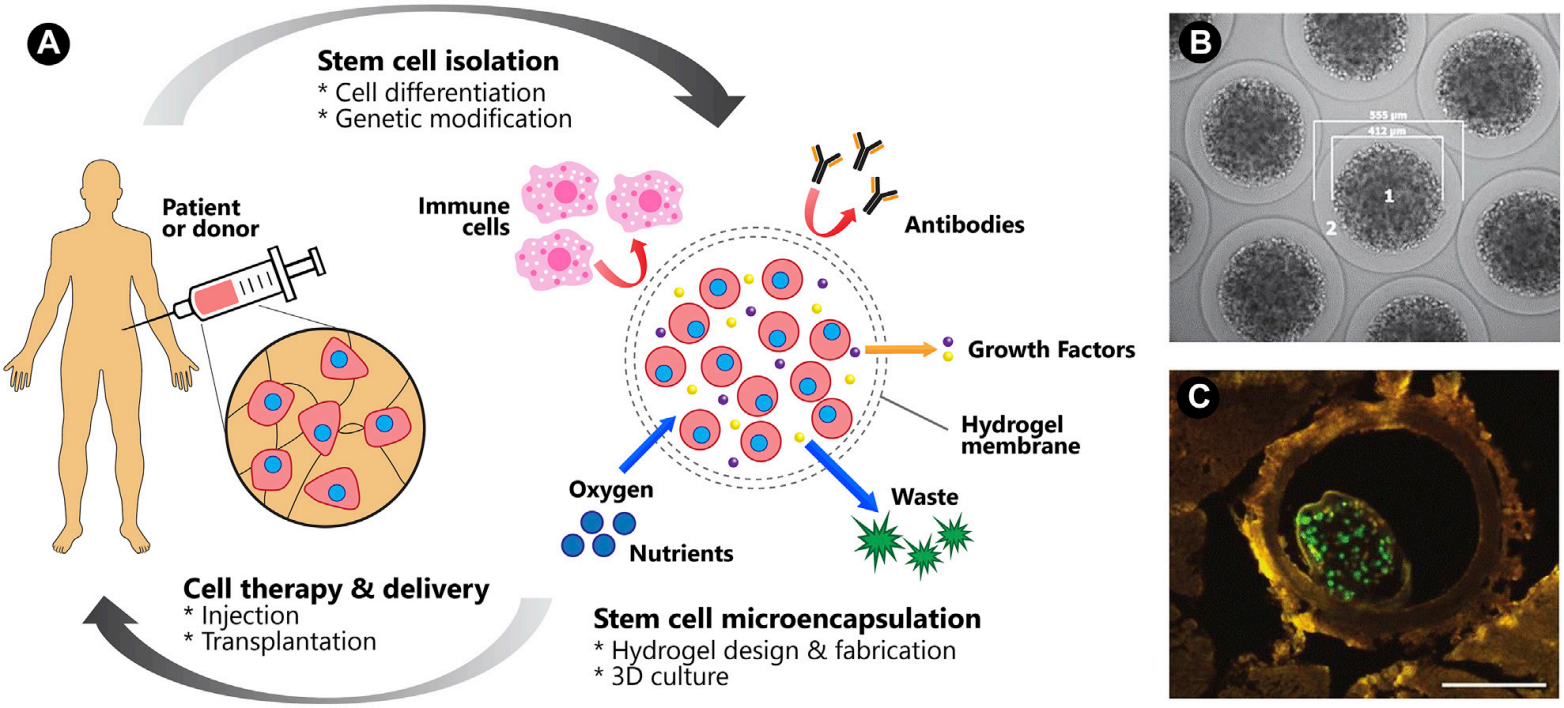
Cell therapy and delivery. **(A)** A schematic of the microencapsulation of human stem cells and its benefits for clinical translation. Figure adapted with permission from Figure 1 of (Choe et al., 2018). **(B)** GLP-1 CellBeads illustrating the central core bead containing GLP-1-secreting MSCs (1) and the surrounding alginate shell (2). **(C)** A green fluorescence protein-tagged CellBead within a coronary vessel. Scale bar: 250 μm. Figures 5B,C adapted with permission from Figures 1A,B of (Wright et al., 2012).

While stem cell therapies have made significant progress preclinically, clinical translation remains challenging due to the massive cell death during transplantation and the failure of the graft to integrate into the host tissue. Hydrogel-based delivery systems emerge as a promising platform to tackle these challenges by preventing mechanical cell damage during cell delivery and creating a favorable microenvironment post-transplantation. Hydrogels derived from natural, synthetic materials or a hybrid of both have been engineered with desirable features. The design of injectable hydrogels allows for localized cell delivery in a minimally invasive manner. Moreover, the ability of hydrogel systems to co-deliver bioactive molecules and to be modified further, enhanced the therapeutic effects (Youngblood et al., 2018) (Figure 5). In this section, the recent progress of polysaccharide-based hydrogels for cell delivery in the musculoskeletal, cardiac, neural and cancerous tissues towards clinical translation is reviewed.

#### Musculoskeletal Tissues

Cell therapies for musculoskeletal tissue restoration are at different levels of evidence in clinical trials. Various sources of stem cells (bone marrow-derived MSCs, umbilical cord-derived MSCs, synovial MSCs, induced PSC, muscle satellite cells) and multiple delivery methods (implantation, arthroscopy, injection) are being explored to optimize the therapeutic effects (Loebel and Burdick, 2018). Modification of hydrogel biophysical properties such as incorporation of integrin-binding motifs has been proven to augment the muscle regeneration. RGD-coupled alginate hydrogel encapsulated gingival mesenchymal stem cells (GMSCs) delivered with multiple myogenic growth factors (containing 6-Bromo-1-methylindirubin-3′-oxime, forskolin, and basic-fibroblast growth factor) was reported to stimulate the expression of myogenetic-related genes and support myogenic differentiation. In animal trial, muscle-like structures were formed in small islands 8 weeks after ubcutaneous transplantation of GMSCs in microbeads into immunocompromised mice (Ansari et al., 2016).

In cartilage repair, the advanced design of a hydrogel system transforms stem cell therapy to the next level with promising clinical application. An alginate (Alg)/polyacrylamide (PAAm) double network (DN) hydrogel system functionalized with transforming growth factor beta-3 (TGF-b3)-encapsulated nanoparticles has been shown to improve the regeneration of cartilage in rats, which is attributed to its physical stability and controlled release of TGF-b3 (Saygili et al., 2021). HA appears as an important component in the hydrogel system. It has been studied in combination with 1) polyethylene glycol and MSCs, namely ChonDux hydrogel, and 2) allogenic human umbilical cord blood derived MSCs, namely Cartistem^®^ for clinical trials (Park et al., 2017; Wolf et al., 2020). Results from the clinical trials suggest that these hydrogel systems are safe and effective for cartilage regeneration to treat cartilage defect in osteoarthritis (Table 2). Despite being highly effective and biodegradable, adverse events such as joint pain, joint effusion and inflammation limit the clinical translation of HA hydrogel systems (Abhirup et al., 2020).

**TABLE 2:** Clinical trials using polysaccharide hydrogels with/without stem cells as of January 2021 (ClinicalTrials.gov).

Polysaccharide hydrogels	Disease/Condition	Intervention/Treatment	Clinical trial	Id number
Hyaluronic acid	Musculoskeletal: Cartilage Defect	Device: ChonDux (combination of HA and PEG encapsulated with MSCs	Terminated (Enrolment suspended; follow up continue)	NCT01110070
Hyaluronic acid	Musculoskeletal: Degeneration Articular Cartilage Knee	Biological: Cartistem (HA hydrogel encapsulated with MSCs derived from allogenic human umbilical cord blood)	Completed phase 2	NCT01733186
Alginate	Neural: Intracerebral Hemorrhage	Drug: GLP-1 CellBeads (alginate microcapsules containing allogenic MSCs, transfected to secrete Glucagon like peptide-1)	Phase 2 (Terminated for improvement of study medication)	NCT01298830
Alginate	Cardiac: Vesicoureteral reflux	Drug: Chondrocyte-alginate gel suspension	Phase 3	NCT00004487
Alginate	Cardiac: Acute Myocardial Infarction; congestive heart failure	Device: IK-5001 (Alginate + calcium gluconate + saline solution	Completed	NCT01226563
Alginate	Cardiac: Dilated cardiomyopathy	Device: Algisyl-LVR	Completed phase 2	NCT00847964
Alginate	Cardiac: Dilated cardiomyopathy; heart failure with reduced ejection fraction	Device: Algisyl-LVR	Completed phase 3	NCT01311791
Alginate	Cardiac: Dilated cardiomyopathy; heart failure	Device: Algisyl	Not yet recruiting	NCT03082508

While hydrogel has exhibited great potential in regenerating many types of musculoskeletal tissues, its application in craniofacial bone tissue repair has been restricted due to its weak adherence to the host tissue. This has been resolved with encapsulating gingival MSCs with an alginate-based, adhesive, photocrosslinkable hydrogel with modifiable mechanical properties. This approach has exhibited satisfying biocompatibility, biodegradability and osteoconductivity in a rat peri-implantitis model (Hasani-Sadrabadi et al., 2020).

#### Cardiac Tissues

Heart failure is among the major causes of death worldwide. Massive cell death in cardiovascular diseases means substantial amount of stem cells are required to reconstitute the cardiac tissue. Co-transplantation of stem cells with hydrogels appeared as one of the appealing strategies to improve both cell delivery and cell survival. A recent study screened the efficacy of matrigel, alginate and hyaluronate as carriers to deliver hESC-derived cardiomyocytes (hESC-CM) using a rat acute myocardial infarction (AMI) model. Although all three delivery systems give rise to improved cardiac function compared with the saline control group, hyaluronate hydrogel is superior in improving cardiac functional recovery, delaying left ventricular remodeling, and preventing arrhythmias (Tan et al., 2020).

Innovative hydrogel designs have emerged to improve the therapeutic efficacies of stem cell therapies via targeted delivery, improved cell retention and increased cell viability (Peña et al., 2018). A bioglass/g-polyglutamic acid/chitosan (BG/g-PGA/CS) injectable composite hydrogel loaded with MSCs has resulted in enhanced cardiac tissue repair in a rat AMI model (Gao et al., 2020). Incorporation of bioactive molecules including cytokines and/or growth factors may increase the efficacy of stem cell therapy by stimulating stem cell proliferation *in vivo* after transplantation. Increase in graft size compared to controls in a rat AMI model has been observed when insulin-like growth factor-1 (IGF-1) was delivered in chitosan hydrogel with human placenta-derived MSCs (Yao et al., 2020). In addition, encapsulation of human vascular endothelial-cadherin (hVE-cad-Fc) fusion protein functionalized MSC aggregates (FMA) using HA-based hydrogel has exhibited better recovery of cardiac function and improved revascularization of infarcted myocardium in comparison to the conventional hydrogel-MSC delivery system (Lyu et al., 2020). In clinical trials, until now, only alginate hydrogels were applied as a device (without cells and bioactive molecules) in treating heart failure, namely IK-5001, Algisyl-LVR and Algisyl (Frey et al., 2014; Lee et al., 2015). Details on promising alginate-based systems in cardiac regeneration and clinical trials can be referred to the current review paper (Giada et al., 2020).

#### Neural Tissues

Delivery of stem cells that can either produce therapeutic molecules to support neural regeneration, or simply substitute the injured or dead cells, is a major paradigm of cell therapy in the management of neural tissue damage (Hlavac et al., 2020). The clinical application potential of various types of hydrogels have been reported. A commercially available HA hydrogel (the HyStem-C Cell Culture Scaffold Kit, BioTime Inc. Alameda, CA, GS313), of which the HA backbone was chemically modified with gelatin to encapsulate and deliver human ESC derived neural stem cells, successfully improved locomotor function in a rat spinal cord injury model (Zarei-Kheirabadi et al., 2020).

Hydrogels provide a 3D microarchitecture that is conducive for neural regeneration in many ways. For example, they provide the physical cues to guide stem cell differentiation, allow the release of bioactive molecules (e.g., brain-derived neurotrophic factor, BDNF) in a controlled manner and shield stem cells from host immune surveillance (Madhusudanan et al., 2020). HA hydrogels functionalized with RGD adhesive peptide and heparin have been demonstrated to promote post-transplantation survival of the highly fragile human ESC-derived midbrain dopaminergic neurons (Vazin and Freed, 2010). In the setting of neurosensory hearing loss, encapsulating BDNF-producing MSCs with ultra-high viscous-alginate has been shown to prevent spiral ganglion neurons from degeneration when applied to the cochlear implant surface in deafened guinea pigs (Scheper et al., 2019). Interestingly, MSCs encapsulated in agarose-carbomer based hydrogel secreted CCL2 chemokine to preserve cytoarchitecture and promote functional recovery in spinal cord injury (Papa et al., 2018). In addition, hydrogel microbeads made of alginate–Ca^2+^were used to differentiate and scale up dopaminergic neurons from encapsulated human iPSC-derived precursor cells. Sufficient cell number was obtained when retrieved for transplantation and the cells were found well integrated with the host brain in pre-clinical study (Komatsu et al., 2015). In clinical trials, allogenic MSCs, transfected to secrete glucagon like peptide-1, were encapsulated in alginate microcapsules (GLP-1 CellBeads). Phase-II results showed no safety issues or adverse events after implantation in the stroke patients with space-occupying intracerebral hemorrhage or traumatic brain injury (Heile and Brinker, 2011).

#### Cancerous Tissues

The role of stem cell therapy in the management of cancer patients is not restricted to replace damaged tissue after cancer treatment (e.g., transplantation of HSCs to facilitate hematological recovery, iPSCs-derived hepatocytes to repair liver tissues), but also involves localized delivery of anti-cancer therapies. Stem cells have been engineered to exhibit tumor-killing properties through the expression of cytotoxic enzymes that process prodrugs (e.g., cytosine deaminase, herpes simplex virus-thymidine kinase), secretory factors (e.g., TNF-alpha-related apoptosis-inducing ligand/TRAIL, IFN-beta) and oncolytic viruses (e.g., herpes simplex virus, HSV). A recent role of stem cells is to carry chemotherapy-containing nanoparticles and protect them from host immune clearance. In cancer immunotherapy, transplantation of HSCs expressing chimeric antigen receptors (CARs) or T-cell receptors (TCR) that are specific for tumor-associated antigen, is a promising strategy to treat hematological malignancies (Zhang et al., 2017). However, this approach is less feasible for solid tumors due to poor infiltration, which could be solved with a localized implantation system. Tumor-targeting CAR T cells and stimulator of IFN genes (STING) agonist delivered by alginate scaffolds has been demonstrated to eradicate solid tumors, which systemic T cell injection alone failed to achieve (Smith et al., 2017). In addition to the implantable form, an injectable hydrogel has shown impressive therapeutic effects as a carrier for cancer vaccine and anti-PD-1 monoclonal antibodies (Li et al., 2020). A recent review covers this area in depth from the hydrogel materials, therapeutic strategies as well as clinical perspectives (Correa et al., 2021).

### Disease Modeling

Apart from its application in cell therapy, biomimetic polysaccharide hydrogels also harbor great potential in disease modeling. However, unlike hydrogels for cell therapy, which emphasize on the homing and engraftment ability, the preferred choice of hydrogels for disease modeling are disease-specific, with emphasis on candidates which carry characteristics that could facilitate the replication of disease phenotypes. Polysaccharide hydrogels have distinct advantages with the ease of modification in mechanical property, permeability, accessibility to nutrients and the ability to imitate the pathophysiological states of various diseases including musculoskeletal, cardiac, neural diseases and cancers.

#### Musculoskeletal Disease

One of the most prevalent musculoskeletal diseases is osteoarthritis (OA), attributed to the degeneration of articular cartilage which involved the loss of chondrocytes. Traditional 2D culture of chondrocytes was found to be suboptimal as chondrocytes dedifferentiated within two passages in such a condition (Aurich et al., 2018). Nevertheless, when chondrocytes were encapsulated in alginate hydrogel in 3D culture condition, chondrocytes maintained their differentiated form (Aurich et al., 2018). It was later discovered that the maintenance of the chondrogenic phenotype was attributed to physical entrapment instead of the chemical interaction with the alginate molecules (Cooke et al., 2017). In addition, chondrocytes encapsulated in alginate methacrylate (ALMA) also exhibited frictional properties, which were similar to stage-3 to stage-4 OA (Meinert et al., 2017). When Meinert and co-workers applied a sliding shear motion on the encapsulated chondrocytes to replicate the mechanical environment of the native cartilage, the chondrocytes responded to the excessive strain by increasing the expression of matrix metalloproteinase-3, which facilitated the degradation of surrounding ECM proteins, resulting in the reduction of stiffness. In the effort to investigate the role of stiffness in the pathogenesis of OA, models of chondrocytes encapsulated in 2% (low stiffness) or 4.5% (high stiffness) agarose to imitate the stiffness of the osteoarthritic or healthy cartilage were created respectively (Jutila et al., 2015). Metabolites were found to be differentially regulated when the low and high stiffness models were subjected to a minimum of 15 min of dynamic compression. Moreover, chondrocytes encapsulated in alginate also responded to various anabolic cues such as connective tissue growth factor (CTGF), bone morphogenetic protein 4 (BMP-4), fibroblast growth factor-2 (FGF-2), insulin growth factor-I (IGF-I), epidermal growth factor (EGF), and Cadherin11 or Matrix Gla Protein (MGP) by increasing the synthesis of glycosaminoglycan (GAG), one of the main proteoglycans that plays a role in maintaining a healthy cartilage structure (Neidlin et al., 2018).

#### Neural Disease

One of the early 3D *in vitro* models of neural tissues was developed by encapsulating neural stem cells (NSCs) in alginate, agarose and carboxy-methyl chitosan via 3D bioprinting, which had allowed *in situ* differentiation of NSCs into functional GABAergic neurons, astrocytes and oligodendrocytes (Gu and Mooney, 2016). The efficient *in situ* differentiation was attributed to the stiffness of the hydrogel, which was maintained at 0.8 kPa, which closely mimicked the stiffness of the human’s brain (Handorf et al., 2015). The stiffness was further reduced to 0.51 kPa by encapsulating iPSC-derived neural progenitors (iPSC-NPC) in the softer methacrylated hyaluronic acid (Me-HA) hydrogel. The soft stiffness had facilitated spontaneous neural differentiation and neurite outgrowth of iPSC-NPC. In addition, it induced expression of neuron-specific proteins in iPSC-NPC derived from Down’s syndrome patients, which otherwise have impaired neurogenesis (Wu et al., 2017). It is important to note that despite many studies having demonstrated the success of 2D cultures in recapitulating the phenotypic hallmarks of various neurodegenerative diseases including amyotrophic lateral sclerosis, Alzheimer’s, Parkinson’s and Huntington’s disease, the majority of studies belong to the familial cases (Centeno et al., 2018). *In vitro* modeling of the more prevalent, sporadic version of such diseases are often more challenging as the replication of disease phenotypes are highly dependent on the physical, chemical and mechanical cues in the microenvironment. A recent study had demonstrated that the amyloid-β plaques formed in an Alzheimer’s disease model may exhibit varying cytotoxicity depending on whether they were confined in 2D or 3D space (Simpson et al., 2020). Agarose, collagen, hyaluronic acid and polyethylene glycol hydrogel cultures were shown to enhance the amyloid-β aggregation towards the larger species which confer lower cytotoxicity as compared to when amyloid-β plaques were found in monolayer cultures. This implies that any future development of Alzheimer’s disease model requires careful consideration concerning the pore size of the hydrogels to recapitulate the physiological condition in the brain.

#### Cardiac Disease

To date, the majority of 3D *in vitro* cardiac disease models were mainly based on cells that were microencapsulated in ECM proteins such as fibrin, gelatin, collagen and Matrigel (Sacchetto et al., 2020). However, ECM protein-based hydrogels suffer from limitations due to batch-to-batch variation, poor mechanical properties and rapid degradation. Integration of polysaccharide hydrogels with ECM proteins could improve the versatility of the resulting hydrogels by offering flexible control over their mechanical properties. For example, gelatin, which has gelation temperature below physiological condition, suffers from poor mechanical properties. When combined with gellan gum, it supported a prolonged culture of cardiomyocytes in 3D. The covalent hydrazone crosslinking of gelatin and gellan gum had allowed the cardiac model to maintain its elasticity, which closely mimicked the native heart for at least 7 days (Koivisto et al., 2019). The state of maturation of the microencapsulated cardiomyocytes, however, was not determined in this study. Maturation of cardiomyocytes is crucial for the accurate modeling of heart diseases and in particular diseases which have late-onset such as heart failures and atrial fibrillation. Composite hydrogels made of hyaluronic acid and collagen had been shown to improve the magnitude of cardiac contraction force (Dahlmann et al., 2013). Moreover, the resulting cardiomyocytes exhibited a well-developed and organized sarcomeric structure that collectively indicated improved cardiac maturation. When microencapsulated cardiomyocytes differentiated from iPSC were co-cultured with endothelial cells and stromal cells in Gly-Arg-Gly-Asp-Ser-Pro (GRGDSP) peptide-coupled alginate hydrogel, they achieved structural maturation after 15 days of culture, as evidenced by the presence of matured myofibril alignment accompanied by elongated and well-organized sarcomeres, which were absent in the control cardiomyocytes aggregates (Abecasis et al., 2020). The resulting model also demonstrated dose-dependent response towards known cardiotoxins such as doxorubicin. Similar dose-dependent toxicity response was also observed in the endothelialized heart-on-a-chip model established by adopting bioprinted alginate-gelatin methacryloyl (GelMA) composite hydrogels, making them useful for drug screening (Zhang et al., 2016).

#### Cancers

**FIGURE 6:**
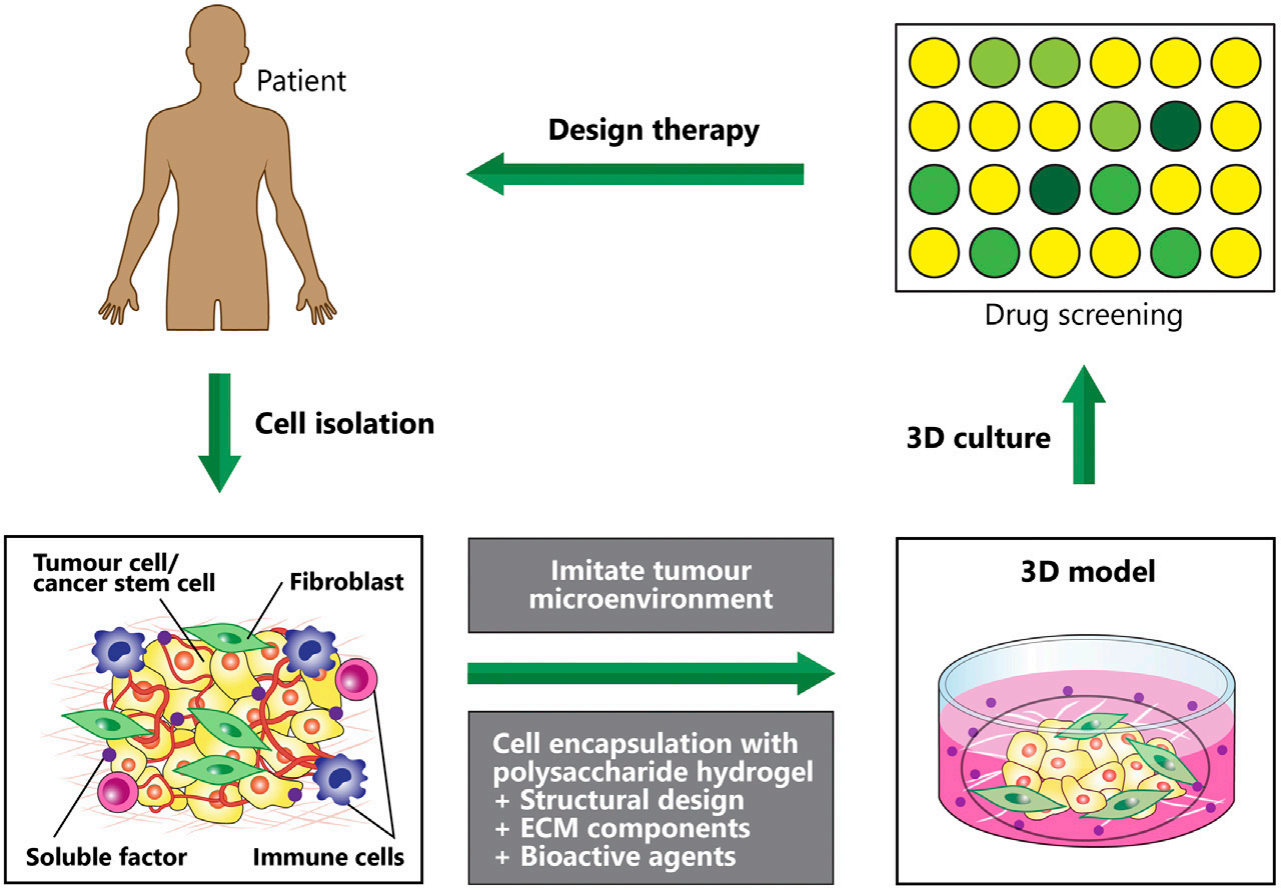
Disease modeling: Creating 3D *in vitro* human tumour models to mimic various microenvironmental cues of human tumours, which can be used to screen anticancer therapeutics. Hydrogel fabrication and cell microencapsulation are involved in engineering the microenvironmental conditions. Impact of each condition on the efficacy of the therapeutic approaches is determined by efficient manipulation and regulation of the microenvironment conditions.

Tumor microenvironments with their complexity, diversity and dynamic nature, play critical roles in cancer development and metastasis (Hanahan and Weinberg, 2011). A current research trend involved the creation of 3D tumor microenvironments recapitulating native tumors by using various engineered polymeric hydrogels (Gu and Mooney, 2016) and stem cells, which enabled the studies of basic cancer biology and screening of the efficacy of anticancer drugs (Roudsari and West, 2016) (Figure 6). At present, 67% of drugs failed phase two clinical trials whilst only 12% completed all stages (Hay et al., 2014). Disease modeling in cancer utilizing natural polymeric hydrogels encapsulated with stem cells has not been widely investigated as compared to other non-communication diseases. *Cancer* stem cells and cancer cell lines are commonly used for encapsulation to mimic the heterogeneity of tumor microenvironment. Recently, 3D bioprinted tumor constructs using modified alginate-gelatin-fibrinogen biomaterials and glioma stem cells were reported to support cell survival, glioma stem cell proliferation, inheritance of cancer stem cell characteristics as well as to exhibit differentiation and vascularization potential (Xingliang et al., 2016; Zhu et al., 2016). When this model tested with temozolomide, higher resistance against temozolomide were found as compared to those in the 2D cell culture model. Another 3D bioprinted hydrogel infused with hydroxyapatite nanoparticles was developed to mimic tumor and bone microenvironments (Zhu et al., 2016). This model served as a tool for exploring cancer metastasis (invasive of breast cancer to bone) and assessing anticancer drug sensitivity. The breast cancer cell spheroids exhibited high migratory ability when co-cultured with bone marrow derived MSCs and demonstrated higher anticancer drug resistance when compared to the 2D culture model.

## Future Perspective and Conclusion

We reviewed studies on stem cell microencapsulation in hydrogels, highlighting the polysaccharide polymers, recent microencapsulation techniques and clinical translation in cell-based therapy (cell delivery) and disease modeling (drug testing and discovery). Despite current promising results and potentials, there are still several challenges and issues remaining. The relatively low viability of encapsulated cells is among the issues. For example, microencapsulation techniques that involve photopolymerization with radial initiators during encapsulation may cause cell damage whilst oil emulsion can destroy the lipid membrane of cells. Thus, current techniques need to be enhanced to reduce the use of radicals, decrease light intensity as well as to limit contact time with the oil phase.

Moreover, most of the techniques are established and tested for stem cells encapsulation at a small scale. Further optimization for large-scale production in the future studies are required to meet the criteria of good manufacturing production (GMP) guidelines. In clinical trials, large quantity of stem cells with approximately 10^7^–10^10^ cells per patient is administered. It is tricky to uniformly encapsulate and expand a large number of cells whilst high cell viability and functionality still remained during and after the 3D culture processes. It is believed that advances in micro-technology and smart material synthesis will help to solve the issues and offer new options for stem cell microencapsulation. The use of a bioreactor in 3D culture and large-scale production may improve cell expansion and cell quality. Importantly, hydrogel materials and processes for stem cell microencapsulation should further modify and customize according to their specific applications in clinical translation as well as display the desired structural, biological, and physicochemical properties. The hydrogel materials must also obtain an approval from regulatory authority such as Food and Drug Administration (FDA) and the equivalent.

To increase the ability of hydrogels and cells to respond to physical, chemical and biological stimuli, diversity in material design is a prospect. Novel synthetic ECM mimetics are suggested to formulate into natural polymers to enable dynamic changes in their properties and reaction to their external environment. Future interest has been drawn to produce ‘smart hydrogels.’ Several types of ‘smart hydrogels’ suitable for stem cell encapsulation and delivery are light responsive hydrogels, electro responsive hydrogels, magnetic responsive hydrogels, pH-responsive hydrogels, glucose responsive hydrogels and biochemical responsive hydrogels (Mantha et al., 2019). Stimuli-responsive hydrogels or cell vehicles could direct migration and cell homing *in vivo* (Wan et al., 2020). Hydrogel materials with programed shape and size are expected to transform post-implantation to fit the defect or transplant site with precise geometry.

Nowadays, advanced hydrogel designs are progressing toward multicomponent and multicellular approaches to increase complexities and heterogeneities in the hydrogel constructs for better tissue integration, sustainable function and effective therapeutic strategy. Multicellular constructs such as stem cell-derived organoids incorporated with polysaccharide hydrogels present the opportunity to create compositionally tailored *in-vitro* tissue models in a high-throughput manner. The models can be utilized in future drug testing and discovery, including toxicological screening and the possibility for drug stratification at a personalized level (when combined with patient-derived iPSCs). It is also foreseen that the reviewed and suggested strategies may potentially apply in precision medicine and personalized tissue regeneration.

The microencapsulation methods discussed in this review are the approaches currently available. Latest and upcoming development such as next-generation 3D bioprinting, namely 4D bioprinting, is believed to provide enormous application in regenerative medicine when moving towards clinical translation. 4D bioprinting could be used for ‘smart hydrogels’ fabrication and advanced stem cell microencapsulation. It offers capability to synthesis shape-programed and functional structured hydrogels in a regulated manner, leading to construction of active multilayered, functional tissues and disease models with dynamic and hierarchical structures (Liaw et al., 2018). Under multiple stimuli, the complex shape transformation processes and functional transitions can facilitate tissue remodeling and maturation.

In conclusion, we detailed various commonly used polysaccharide hydrogels and their unique properties, types of stem cells and current microencapsulation methods with recent studies demonstrating the potential of stem cell-encapsulated hydrogels in cell delivery and disease modeling for treating diseases. While the choice of hydrogel material and design impact the viability and differentiation of encapsulated stem cells, the different microencapsulation techniques have also shown variable cell activities post-encapsulation. There are still many problems to solve when moving towards clinical translation. Future developments are now focusing on the combining of different materials, multiple cell types and more than one microencapsulation technique to work in a complementary mode. The challenges and limitations discussed herein need to be further addressed in future studies to promote the therapeutic activity and applicability of microencapsulated stem cells in regenerative medicine.

## Footnotes

1 ESCs: Embryonic Stem Cells; iPSCs: induced Pluripotent Stem Cells; MSCs: Mesenchymal Stem Cells; ASCs: Adipose-derived Adult Stem Cells
